# Supplementary material for: Resolving the full spectrum of human genome variation using Linked-Reads
Source: Genome Res. 2019 Apr;29(4):635–45. doi: 10.1101/gr.234443.118 (PMC6442396; doi:10.1101/gr.234443.118)
Supplement: Supplemental Material [file supp_gr.234443.118_Supplemental_longranger-2.2.zip › longranger-2.2.tar.bz2!/lr2.2-public/lib/stan/cmdstan/stan/doc/api/html/univariate_2continuous_8hpp_source.html]

Stan: src/stan/prob/distributions/univariate/continuous.hpp Source File


|  |  |
| --- | --- |
|  | Stan  2.5.0  probability, sampling & optimization |


- Main Page
- Related Pages
- Namespaces
- Classes
- Files

- File List
- File Members

All Classes Namespaces Files Functions Variables Typedefs Enumerations Enumerator Friends Macros Pages

- src
- stan
- prob
- distributions
- univariate

continuous.hpp

Go to the documentation of this file.

1 #ifndef STAN\_\_PROB\_\_DISTRIBUTIONS\_\_UNIVARIATE\_\_CONTINUOUS\_HPP

2 #define STAN\_\_PROB\_\_DISTRIBUTIONS\_\_UNIVARIATE\_\_CONTINUOUS\_HPP

3

4 #include <stan/prob/distributions/univariate/continuous/beta.hpp>

5 #include <stan/prob/distributions/univariate/continuous/cauchy.hpp>

6 #include <stan/prob/distributions/univariate/continuous/chi\_square.hpp>

7 #include <stan/prob/distributions/univariate/continuous/double\_exponential.hpp>

8 #include <stan/prob/distributions/univariate/continuous/exponential.hpp>

9 #include <stan/prob/distributions/univariate/continuous/exp\_mod\_normal.hpp>

10 #include <stan/prob/distributions/univariate/continuous/frechet.hpp>

11 #include <stan/prob/distributions/univariate/continuous/gamma.hpp>

12 #include <stan/prob/distributions/univariate/continuous/gumbel.hpp>

13 #include <stan/prob/distributions/univariate/continuous/inv\_chi\_square.hpp>

14 #include <stan/prob/distributions/univariate/continuous/inv\_gamma.hpp>

15 #include <stan/prob/distributions/univariate/continuous/logistic.hpp>

16 #include <stan/prob/distributions/univariate/continuous/lognormal.hpp>

17 #include <stan/prob/distributions/univariate/continuous/normal.hpp>

18 #include <stan/prob/distributions/univariate/continuous/pareto.hpp>

19 #include <stan/prob/distributions/univariate/continuous/pareto\_type\_2.hpp>

20 #include <stan/prob/distributions/univariate/continuous/rayleigh.hpp>

21 #include <stan/prob/distributions/univariate/continuous/scaled\_inv\_chi\_square.hpp>

22 #include <stan/prob/distributions/univariate/continuous/skew\_normal.hpp>

23 #include <stan/prob/distributions/univariate/continuous/student\_t.hpp>

24 #include <stan/prob/distributions/univariate/continuous/uniform.hpp>

25 #include <stan/prob/distributions/univariate/continuous/von\_mises.hpp>

26 #include <stan/prob/distributions/univariate/continuous/weibull.hpp>

27

28

29 #endif

chi\_square.hpp

student\_t.hpp

beta.hpp

pareto.hpp

uniform.hpp

pareto\_type\_2.hpp

weibull.hpp

normal.hpp

double\_exponential.hpp

exp\_mod\_normal.hpp

rayleigh.hpp

scaled\_inv\_chi\_square.hpp

lognormal.hpp

skew\_normal.hpp

gumbel.hpp

inv\_chi\_square.hpp

logistic.hpp

exponential.hpp

inv\_gamma.hpp

cauchy.hpp

frechet.hpp

von\_mises.hpp

gamma.hpp


---

[ Stan Home Page ]

*© 2011–2014,
Stan Development Team.*
